# A Curious Case of Fracture above the Third Vertebra Cervicalis

**Published:** 1854-02

**Authors:** 


A Curious Case of Fracture above the Third Vertebra Cervicalis.—James W. Claiborne, M. D., of California, relates the following:—Daniel Edson, of Fall river, Massachusetts, aged twenty-seven years, fell from a stage about the 10th day of July 1853. The horses were running at full speed, and as he fell from the driver's seat, he thinks he struck his neck on the tongue or pole of the vehicle.. Reaction was slow in establishing itself, and there was such a cadaverous pallor and collapse, that in connection with other symptoms, his recovery was regarded as hopeless. He maintained a horror of moving from one side to another, was speechless, and desired to be kept in a state of absolute rest for a week or ten days. As soon as the neck was examined, a hard protuberance of osseous firmness was perceived at the back of the neck, on a level with the lobulus pendulus of the ear. It was situated a little to the left of the spinous processes, and under the tendinous origin of the trapezius. It has never increased or diminished in size, and is about the size, of a split walnut. The protuberance is situated in the line of the hair, below which, according to Velpeau, (Anatomy, vol. i, p. 203,) the atlas is seldom situated. It is therefore fair to say, the tumor is above the third cervical vertebra.

There was no difficulty in the breathing, or injury to the phrenic nerve, as might be supposed. The inferior maxilla was for several weeks firmly closed, and it was almost impossible to introduce a spoon between the teeth. The attempt would cause him great pain at the site of the node or protuberance, and an apprehension of danger in the effort. As soon as he was so far recovered as to attempt walking, for many days he would carry his head steadied with a hand over each ear, and at present, some three months after the occurrence, he has but few of the motions of the head. The motion backwards is impossible, and it is only very recently that he has attempted to look down at his feet. With regard to rotation, he turns the head easily towards the tumor which is on the left side of the spine, but it causes him much pain to turn toward the right. Swallowing was greatly interfered with, and for some time after the event he was often troubled with a feeling of there being a small substance lodged in the pharynx. He further informs me that some few weeks ago he received a blow or jar upon the crown of the head, and that he felt a grating or crepitation a little below the occipital protuberance of the right side at that time.—*Va. Med. Gaz.*

